# Decoding Gastric Reflexes: The Role of Mechanosensitive Enteric Neurons in Stomach Motility

**DOI:** 10.1111/nmo.70011

**Published:** 2025-03-03

**Authors:** Gemma Mazzuoli‐Weber, Sophia Mayr, Kristin Elfers

**Affiliations:** ^1^ Institute for Physiology and Cell Biology University of Veterinary Medicine Hannover Hannover Germany; ^2^ Center for Systems Neuroscience (ZSN) Hannover Germany

**Keywords:** enteric nervous system, gastric reflexes, Guinea pig, mechanosensitivity, motility, stomach

## Abstract

This review focuses on mechanosensitive enteric neurons (MEN) in the guinea pig stomach and their roles in gastric motor reflex pathways. The guinea pig model is advantageous for studying gastric physiology, as its stomach structure and function closely resemble those of humans. Gastric motility involves distinct functional regions: the fundus and proximal corpus act as reservoirs, while the distal corpus and antrum handle food mixing and propulsion. Mechanosensitivity in both gastric cholinergic and nitrergic enteric neurons plays a critical role in adapting muscle activity in response to gastric content volume. These neurons enable reflex circuits involved in the accommodation reflex, with cholinergic excitatory and nitrergic inhibitory pathways promoting relaxation. This review summarizes the anatomical, functional, and neurochemical characteristics of MEN across gastric regions, their direct and indirect interactions with smooth muscle, and the role of distinct neurotransmitters in modulating gastric motility. The need for future studies on mechanosensitive pathways and involved neuronal receptors is highlighted to enhance our understanding, finally aiding therapeutic development.

## Graphical abstract

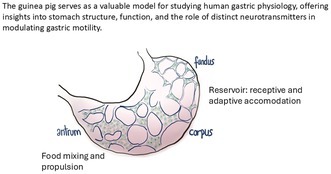

**Summary**

- The stomach of monogastric species is functionally divided into the fundus and proximal corpus acting as reservoirs and the distal corpus and antrum handling food mixing and propulsion.
- Mechanosensitive enteric neurons (MEN) have been identified in all stomach regions and play a critical role especially in mediating the accommodation reflex.
- Gastric neurons, including MEN, use several neurotransmitters such as ACh, NO, and SP, for mediating the distinct motility patterns.
- At least some of the gastric MEN display a multifunctional pattern, directly communicating with the gastric smooth muscle cells.

## 1 The Guinea Pig as a Model to Study the Human Stomach

In monogastric species, the stomach is a part of the digestive tract specialized in the storage and the mechanical and chemical digestion of food, particularly of proteins and fat. Functionally, the monogastric stomach is divided into the fundus and the proximal third of the corpus, serving as a reservoir for the incoming food [1, 2, 3, 4] and the distal corpus and the antrum, mainly responsible for grinding, mixing, and transporting the content further into the duodenum [5]. In order to fulfill these functions, different motility patterns are present in the described distinct gastric regions. However, the underlying regulatory (fine‐tuning) mechanisms are only incompletely understood. This is a critical point, as the number of human patients suffering from gastric dysfunctions and related motility disorders, such as functional dyspepsia (FD) and gastroparesis, has increased worldwide over the last few decades [6].

Recently, functional imaging techniques have been adopted to better investigate and understand gastric motility reflex circuits in human subjects in vivo [7, 8]. However, in vitro examination of fresh and hence vital gastric tissue preparations is indispensable to investigate underlying mechanisms on a cellular level. Since access to such fresh gastric tissue samples from human patients is limited due to ethical reasons, animal models are an important and useful tool. Rodents are commonly used animal models in gastrointestinal research, each offering different advantages. Mice, for instance, can be quite easily genetically modified, with many transgenic models available to study specific aspects of gastric function or disease. However, the gastric anatomy and physiological processes in mice differ more significantly from humans, making them less ideal for studying human‐specific stomach conditions. For instance, the presence of a nonglandular forestomach in mice without secretory activity, which is absent in humans [9], or large differences in gastric emptying rates and pH [10]. The guinea pig's gastrointestinal tract represents a good model for humans for several reasons: the anatomical and functional divisions of its stomach are quite similar to those of humans. Guinea pigs have only glandular stomachs with a cylindrical epithelium. The gastric pH and its regional difference, with higher values in the fundus than in the antrum, are similar to the situation in humans [11, 12, 13]. Additionally, exhibiting a crepuscular activity pattern, rather than a nocturnal one like mice and rats, with peaks during the beginning and end of the light period and a significantly greater amount of feed intake during the light than during the dark period [14]; even though this differs from the (planned) meals with intermittent periods common for humans, it is clearly advantageous compared to strictly nocturnal species like mice and rats. In both species, humans and guinea pigs, the interstitial cells of Cajal (ICCs) act as intrinsic pacemakers for regular gastric contractions [15, 16]. Furthermore, the gastric enteric nervous system (ENS) shows a comparable structure in guinea pigs and humans, with submucosal neurons only sporadically present, except for the human antrum, where a slightly more developed submucosal network can be found. In both species, acetylcholine (ACh) represents the main excitatory neurotransmitter and nitric oxide (NO) the main inhibitory one (mostly in combination with the accessory transmitter vasoactive intestinal peptide (VIP)). The neuronal populations expressing these neurotransmitters are present in comparable proportions in guinea pigs and humans [17, 18, 19, 20, 21].

## 2 Stomach Regions and Motility

The retention period of the ingesta in the stomach varies depending on many factors, including the biochemical properties and the physical structure, as well as the quantity of food present in the stomach and in the duodenum (gastrointestinal feedback). Due to the mentioned functional compartmentalization, the motility of the stomach is complex and quite different from the other regions of the gastrointestinal tract. Briefly, tonic and phasic contractions can be distinguished, either lasting for several seconds to minutes with a slow increase in pressure [22] or lasting for only a few seconds, resulting in a rapid increase in intragastric pressure. The fundus, to fulfill the task of a reservoir, needs to be able to relax and accommodate and hence shows mainly tonic muscle activity. The proximal part of the corpus, similar to the fundus, plays a role in the accommodation reflex, whereas the more distal part of the corpus is important for propulsion and retropulsion and for mixing the stomach contents, and it exhibits both tonic and phasic muscle activity. The antrum strongly mixes and crushes its contents and empties food particles into the duodenum: here, phasic muscle activity clearly dominates. Stomach motility, as well as gastrogastral and gastrointestinal reflexes, are complex and controlled by an interplay between the intrinsic input by the ENS and innervation by extrinsic, mainly vagal afferent and efferent fibers. The main players are, in this context, sensory, inter‐ and motoneurons, ICCs, and smooth muscle cells. Gastric smooth muscle activity is controlled by both neurogenic and myogenic mechanisms [23]. Myogenic activity, defined as the inherent ability of muscle contraction without external neuronal input, is driven by the intrinsic pacemaker cells, the ICCs. They form an electrically coupled network in the myenteric plexus (ICC‐MY) and intramuscularly (ICC‐IM). ICCs are electrically coupled with smooth muscle cells and generate inward calcium currents, resulting in propagating, slow changes in membrane potentials, defined as slow waves [24, 25, 26, 27]. At the maximal amplitude of such slow waves, an additional excitatory input (such as neuronal input) is able to depolarize the smooth muscle cell, finally resulting in muscle contraction. In the stomach, successive descending gastric contractions are triggered by slow waves, which originate in the corpus. These waves occur regularly (3 or 5 per minute in the guinea pig and in the human stomach, respectively) and in the antrum slowly conduct in the anal direction but more rapidly in the circumferential direction [28]. Neurogenic control of muscle activity is mediated by enteric neurons: Excitatory enteric motoneurons depolarize smooth muscle cells either directly via ACh acting on muscarinic receptors, together with tachykinins as accessory transmitters, or indirectly via ICCs. Enteric inhibitory motoneurons mediate muscle relaxation directly by releasing NO, adenosine triphosphate (ATP), VIP, or indirectly via ICCs. Most data indicate that an intact myenteric plexus is required for the coordination of gastric motility and emptying [29, 30, 31].

## 3 The Gastric Myenteric Plexus

**FIGURE 1:**
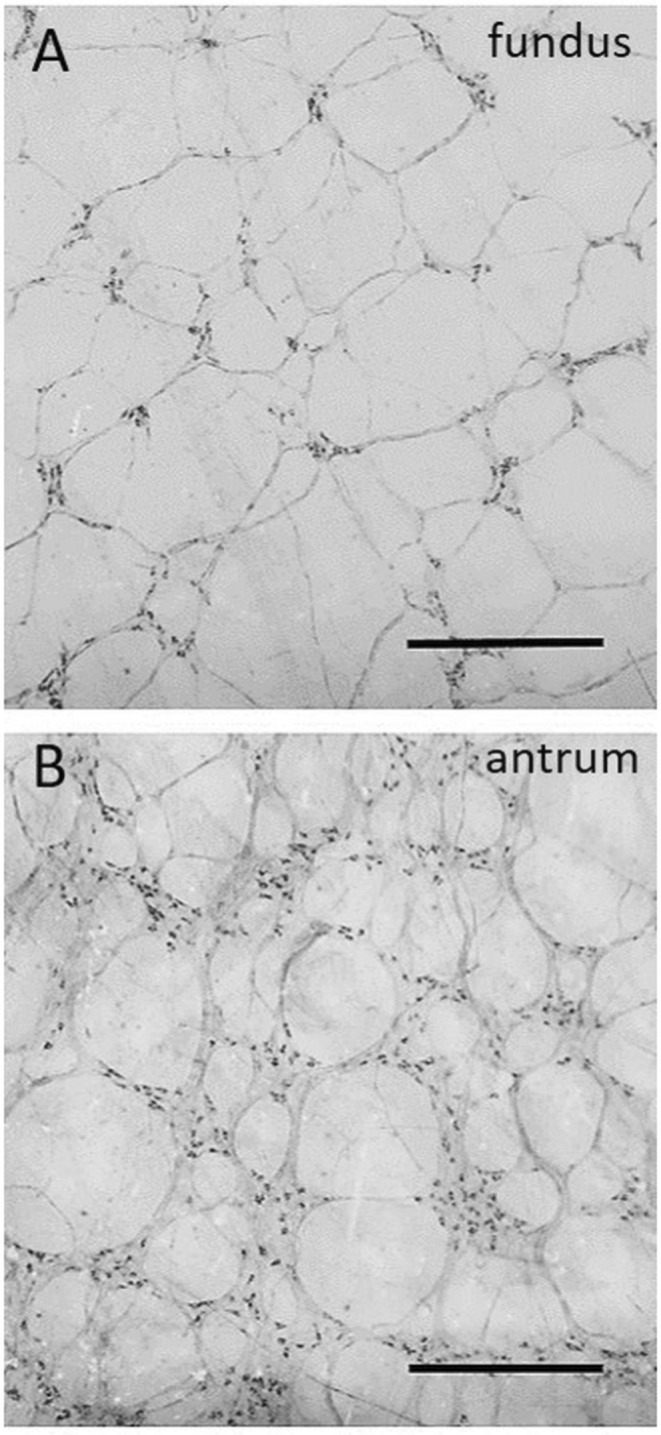
Regional differences in the appearance of the myenteric plexus ganglia of the guinea pig stomach. Staining of myenteric ganglia for nicotinamide adenine dinucleotide phosphate‐diaphorase (NADPH) in the proximal and distal stomach: In the fundus (A) the myenteric ganglia are very small in size and sparsely distributed, creating a loosely organized network. In the antrum (B), the ganglia are huge in size and densely packed. Scale bar = 100 μm.

Within the guinea pig stomach, the myenteric plexus shows a different appearance and number of neurons per ganglion in the distinct gastric regions. In the fundus and near the cardiac orifice, the ganglia are small and elongated, typically containing an average of five to ten neurons per ganglion. Conversely, in the antrum, the ganglia are very large and densely packed [32]. The ganglia in the fundus are sparsely distributed and isolated with few, long interganglionic fibers, whereas in the antrum, they are connected with numerous short interganglionic fibers (Figure 1). An average neuronal count of 3500 neurons per cm^2^ is reported in the fundus, compared with more than 20,000 neurons per cm^2^ in the antrum of the guinea pig [32].

## 4 Neuronal Populations in the Stomach

The enzymes required for the synthesis of the main neurotransmitters ACh and NO are choline acetyltransferase (ChAT) and neuronal nitric oxide synthase (nNOS), respectively. Immunohistochemical staining for these enzymes identifies enteric neurons immunoreactive (IR) for ChAT and nNOS and allows for a neuronal categorization, since a colocalization of both neurotransmitters does almost not occur in gastric myenteric neurons [33, 34]. Studies revealed that in the guinea pig stomach, 64% of ChAT‐IR myenteric neurons were motoneurons, 27% nonmotoneurons, and 9% multitarget neurons, whereas of the NOS‐IR myenteric neurons, 57% were motoneurons, 39% nonmotoneurons, and 4% multitarget neurons [33]. A more precise categorization can be made based on the coexpression of further transmitters such as enkephalin, substance P (SP), neuropeptide Y, VIP, serotonin (5‐HT), calretinin, or calbindin [35]. Multiple studies in the guinea pig stomach reported specific neurochemical coding for myenteric neurons, depending on their respective targets such as mucosa, longitudinal, or circular muscle layer [35, 36, 37, 38, 39, 40]. The myenteric neurons display a region‐specific diversity in the expression of the abovementioned neurotransmitters (Table 1). Contrary to mice and humans [41, 42], in myenteric neurons from the guinea pig, no single‐cell RNA sequencing has been conducted so far. Therefore, a more detailed molecular characterization is still pending.

**TABLE 1:** Neurotransmitters in the guinea pig stomach. Several studies investigated neurotransmitters in the myenteric plexus of the different regions in the guinea pig stomach [5, 6, 33, 34, 37, 38, 39]. Neurons projecting to the mucosa are referred to as mucosa neurons, and neurons projecting to the muscle layers are referred to as muscle motoneurons. Neurotransmitters, which were not investigated in the respective region, are marked as not investigated (n.i.). Depending on the study, the proportions were not always given for the total number of neurons in the myenteric plexus, but as subpopulations as noted [59].

	Fundus	Corpus	Antrum	Targets/Functions
ChAT	57% of all myenteric neurons	67%	56%	
	79% of mucosa neurons	71% of mucosa neurons		Mucosa
	69% of muscle motoneurons			CML and LML
nNOS	45% of all myenteric neurons	29%	40.7%	
	21% of mucosa neurons	28% of mucosa neurons		Mucosa
	31% of muscle motoneurons			CML and LML
SP	6.4% of ChAT‐IR mucosa neurons	33%	37.4%	Excitatory function on smooth muscle layer
	24.8% of ChAT‐IR muscle motoneurons [37]			
VIP	n.i.	38%	21.7%	At least some function as inhibitory motoneurons
NPY	66.7% of nNOS‐IR neurons	38%	28.6%	Unclear
	4% of ChAT‐IR neurons			
5‐HT	n.i.	2%	3.9%	Unclear, might mediate sEPSPs
CALRET	9% of all ENK‐IR neurons	6%	6.8%	At least in parts functioning as motoneurons
CALB	12%/4%	n.d./ 12%/4%	0.5%/25%/16%	Unclear, if functioning as sensory neurons
ENK	44%	55%	n.i.	in fundus primarily projecting to smooth muscle layers, not mucosa

Abbreviations: 5‐HT, 5‐hydroxytryptamine; CALB, calbindin; CALRET, calretinin; ChAT, choline acetyltransferase; CML, circular muscle layer; ENK, enkephalin; LML, longitudinal muscle layer; MP, myenteric plexus; n.d., not detected; nNOS, neuronal nitric oxide synthase; NPY, neuropeptide Y; sEPSP, slow excitatory postsynaptic potential; SP, substance P; VIP, vasoactive intestinal peptide.

The electrophysiological behavior of gastric myenteric neurons has been extensively investigated in the guinea pig with intracellular recording methods [43, 44, 45, 46]. These studies showed that gastric neurons differed significantly from their intestinal counterparts. With regard to their electrophysiological properties, three neuronal subpopulations have been identified in the corpus: Gastric I, II, and III neurons. Gastric I neurons are characterized by repetitive spike discharge during depolarizing current pulses and by higher input resistance than the other types. Gastric II neurons discharged one or two spikes only at the onset of long‐lasting depolarizing current pulses. Gastric III neurons did not discharge spikes to depolarizing current pulses and had higher membrane potentials and lower input resistances than the other types [43]. In the antrum also neurons with long‐lasting hyperpolarizing after‐potentials (AH/Type 2) were also identified [46]. Most gastric neurons receive fast excitatory postsynaptic potentials (fEPSPs). Slow EPSPs have not been recorded in the corpus, while they were present in 14% of the antral neurons (mostly AH/Type 2) [45]. Inhibitory postsynaptic potentials (IPSPs) have not been recorded [44] in the corpus and rarely in the antrum [45]. Thus, the differences in the electrical and synaptic behavior are likely to reflect adaptations connected with the specialized digestive functions of the different gastric regions.

## 5 Accommodation Reflexes

**FIGURE 2:**
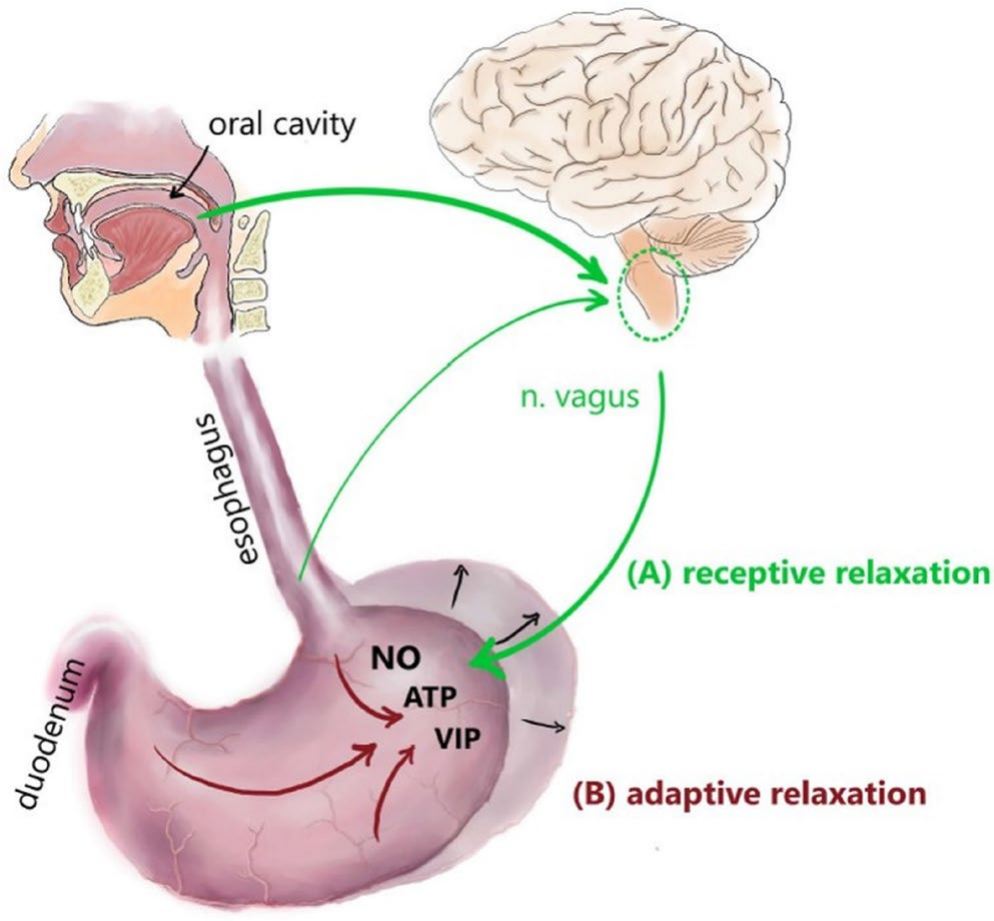
Schematic depiction of the reflex circuits involved in gastric accommodation. The figure exemplarily shows the situation in humans, but accounts for all mammals with single compartment stomachs, including the guinea pig. Mechanical stimulation in the oral cavity, pharynx, and esophagus leads to receptive relaxation in the proximal stomach (A). This extrinsic reflex circuit includes vagal afferent fibers projecting to the medulla oblongata as well as efferent fibers connecting to myenteric inhibitory motoneurons. These, in turn, release inhibitory neurotransmitters, such as NO, ATP, and VIP, leading to a relaxation of the smooth muscle. With further food ingestion, the adaptive relaxation as the second part of the accommodation starts, leading to further dilation of the proximal stomach (B). This intrinsic reflex circuit is triggered by the activation of mechanoreceptors and chemoreceptors within the stomach. The activation of the inhibitory reflex circuits within the enteric nervous system leads to the release of NO, ATP, and VIP, thus to further relaxation of the smooth muscle layers [59]. ATP, adenosine triphosphate; NO, nitric oxide; VIP, vasoactive intestinal peptide.

The (proximal) stomach is able to accommodate even before the ingesta enter. The involved reflexes seem to be highly conserved since they are present in all mammals with single‐compartment stomachs, including humans, pigs, dogs, cats, rats, mice, and guinea pigs, with some differences in the volume change of the proximal stomach in species that ingest brief, large meals, such as cats and dogs [3]. Figure 2 depicts the involved reflex circuits in humans. Conditioned and unconditioned stimuli (e.g., smelling or seeing tasty food) initiate this reflex. Extrinsic vagal afferent and efferent fibers control the initial receptive relaxation of the fundus. Vagal afferent fibers conduct the information toward the central nervous system, from where vagal efferent fibers connect via nicotinic synaptic transmission to myenteric inhibitory motoneurons, therefore using the ENS as a relay station. Such inhibitory motoneurons release neurotransmitters, mainly NO, mediating smooth muscle relaxation. This vagovagal reflex has been described in detail in rodents and in humans [47, 48].

With ingesta entering the stomach, leading to an increase in the intragastric volume, a further accommodation reflex is initiated: the adaptive relaxation. Since it has been shown in various species that this reflex is still present in isolated stomach preparations, it was concluded that it is mainly dependent on the ENS [4, 47, 49, 50]. It is triggered by mechanosensors located in the oral part of the stomach, which communicate with nitrergic motoneurons mediating smooth muscle cell relaxation via mainly NO. Even though the adaptive relaxation is predominantly regulated by the ENS, nevertheless, vagotomy leads to a substantially reduced relaxation, emphasizing that vagal nerve fibers at least play a modulatory role [51, 52].

## 6 Emptying Reflexes

Motility in the distal stomach is characterized by circumferential contractions starting from the pacemaker region in the proximal corpus and moving distally. As soon as the contractions reach the middle antrum, liquids and smaller particles are propelled through the pylorus into the duodenum. Larger particles are thrown back into the corpus due to the strong contraction diminishing the volume of the entire antrum.

While the proximal stomach contributes to gastric emptying by exerting tonic contractions and therefore creating a stable pressure on the content, the distal stomach exerts phasic contractions. These circular contractions occur at a mean frequency of three contractions/min in the human corpus and antrum [8]. For the guinea pig stomach, such propagating peristaltic contractions have only been described in in vitro studies. Here, the contractions were initiated by either rapid filling of the stomach or by electrical vagal stimulation [4, 53]. Both described a similar frequency of 5–6 contractions/min. The basis of those gastric contractions is the already described slow waves, originating from the ICCs located in the pacemaker region [54, 55].

## 7 Mechanosensitive Enteric Neurons in the Guinea Pig Stomach

Enteric neurons capable of perceiving mechanical stimuli and directly mediating to the muscles by releasing different neurotransmitters are called mechanosensitive enteric neurons (MEN). MEN have been identified in all regions of the stomach and by blocking nicotinic synaptic transmission using hexamethonium, it was shown that they are indeed directly activated by the applied mechanical stimuli. MEN can display various neurochemical phenotypes and are present in different proportions in the three gastric regions: 16%, 27%, and 6% of the neurons per ganglion have been identified as MEN in the fundus, corpus, and antrum, respectively [56, 57]. The chemical phenotypes of MEN do also differ depending on the gastric compartment. In the corpus, 55% of MEN are cholinergic and 45% are nitrergic; in the fundus, 83% of MEN are cholinergic, and 19% are nitrergic, whereas in the antrum, MEN are equally distributed among the cholinergic and nitrergic populations. Differently from intestinal MEN, the responsiveness of gastric MEN is affected by desensitization of transient receptor potential vanilloid channel 1 (TPRV1) expressing visceral afferents by long‐term perfusion with capsaicin [56, 57, 58]. This indicates that the mechanosensitivity of gastric MEN is modulated by the crosstalk between intrinsic and extrinsic innervation. It could be speculated that this signaling is at least partially mediated through tachykinins, likely neurokinin 3 (NK3). However, the perfusion with the NK3 antagonist SR142801 was effective in reducing the firing of MEN only in the corpus and not in the antrum, hinting toward region‐specific effects of NK3 [57].

The involvement of other putative neuronal receptors in the mechanotransduction of gastric myenteric neurons in the guinea pig, such as the Piezo1 proteins or the TRPV2, which are involved in gastric accommodation in the mouse stomach, has been excluded by respective experiments [59, 60, 61, 62].

Gastric MEN, in particular in the fundus, appear to have different activation thresholds; in particular, at a higher level of ganglionic stretch, more NOS‐IR MEN are activated [63]. This is consistent with their possible role in coordinating the progressive relaxation in response to volume increase in this compartment during the adaptive relaxation reflex.

## 8 Circuits Involving MEN

**FIGURE 3:**
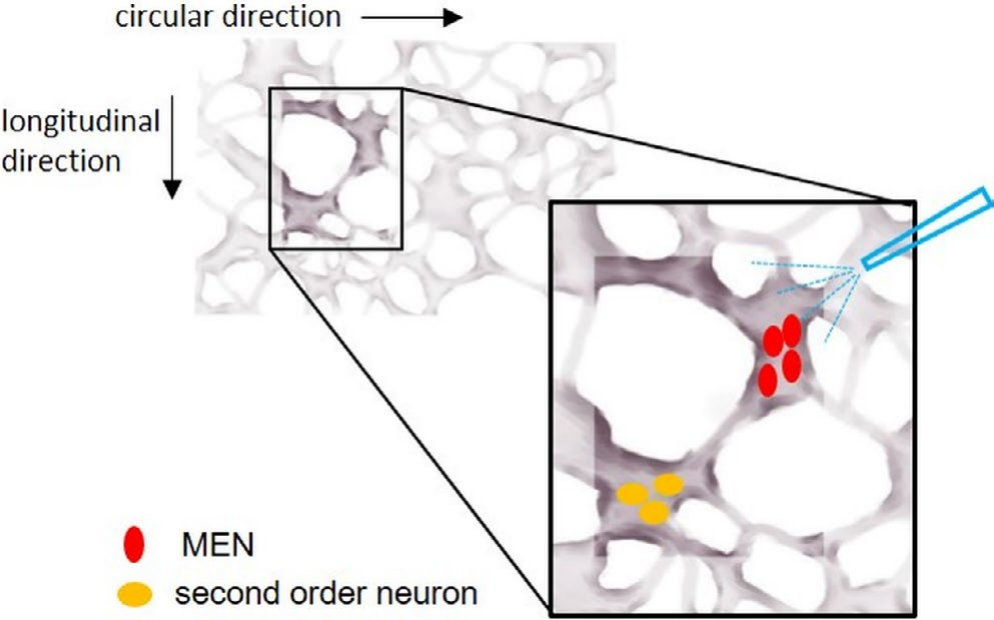
Experimental settings for detecting responses of second‐order neurons. Mechanosensitive enteric neurons (MEN; in red) directly activated by a mechanical stimulus are supposed to send signals activating second‐order neurons (in yellow) located in the neighboring ganglia. The blue pipette depicts the direction of the incoming mechanical stimulation from the primarily stimulated ganglion [59].

**FIGURE 4:**
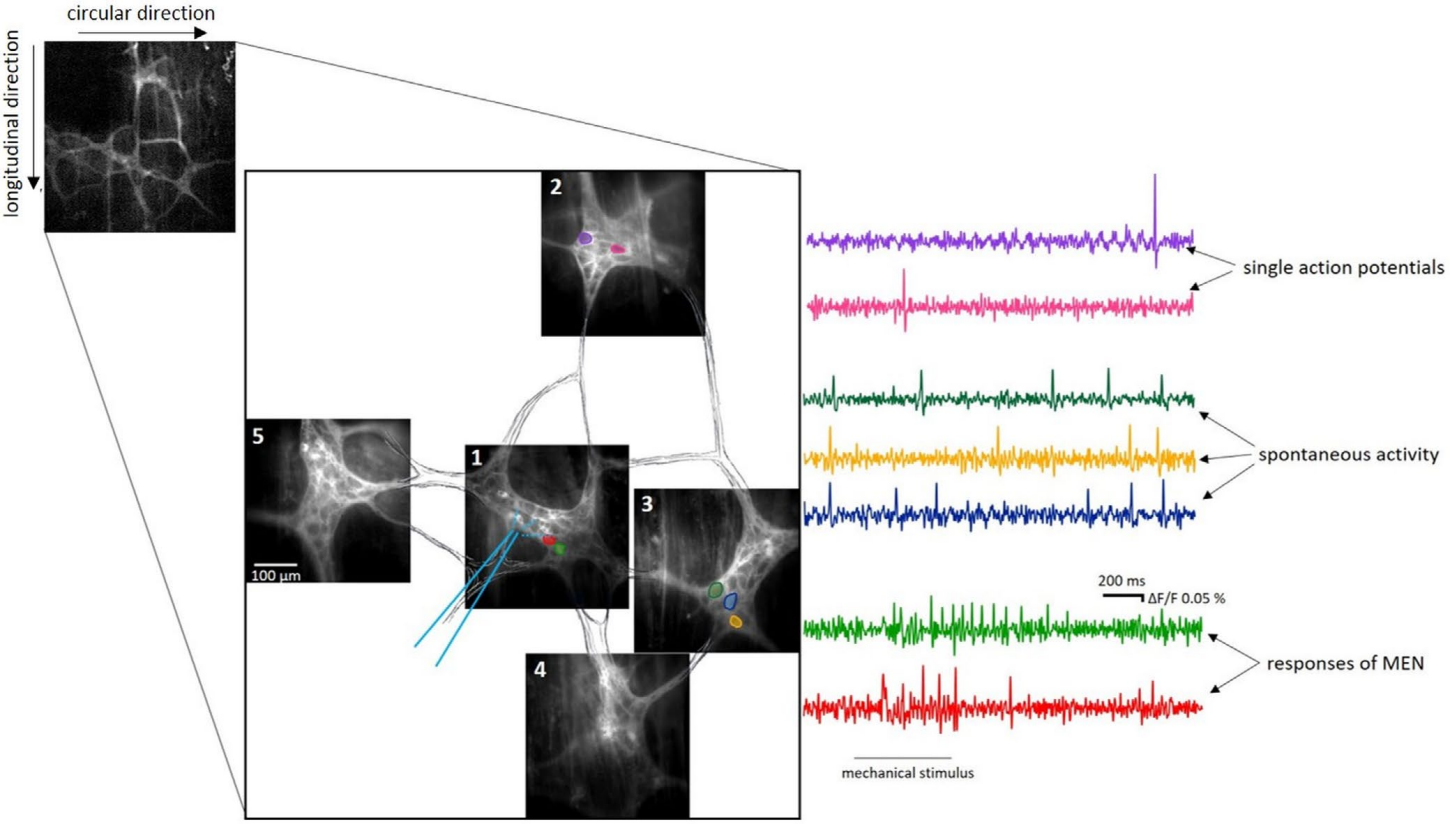
Recording from neighboring ganglia in the fundus. As indicated with the light blue pipette, the ganglion in the middle (1) was stimulated by a small‐volume intraganglionic injection. Here, two exemplary mechanosensitive neurons directly responded to the mechanical stimulus (two traces at the bottom). In the ganglion situated in the ascending direction (2), two neurons discharged single action potentials. Neurons in the connected ganglion lying in the circular muscle direction toward the greater curvature (3) were highly spontaneously active since their action potential discharge already started before the application of the mechanical stimulus in the first ganglion. In two other connected ganglia (4 and 5), no neuronal activity could be detected [59].

The question of whether MEN directly communicate with smooth muscle cells or indirectly through second‐order neurons has been deeply investigated by our group. We performed a large number of experiments aiming to record neuronal activity synaptically transmitted from activated MEN in a certain ganglion to neurons located in neighboring ganglia, referred to as second‐order neurons (Figure 3). In case of recordable responses in second‐order neurons, we blocked cholinergic neuronal communication by hexamethonium. However, in both regions, fundus (Figure 4) and antrum, we only recorded a few reproducible responses in the adjacent ganglia, mostly located in the ascendant direction [59]. Thus, in general, we could not confirm the projection of MEN to second‐order neurons. This might have different reasons: Firstly, we showed that in the guinea pig ileum, MEN can be multifunctional, meaning that they act as sensory and motoneurons, directly projecting to the muscle layers and not to second‐order neurons [58]. Secondly, the adaptive relaxation, as an intrinsic reflex, is independent of nicotinic synaptic transmission [64]. Thirdly, studies on the different projections of cholinergic and nitrergic myenteric neurons in the guinea pig stomach revealed that out of the ChAT‐IR neurons, 64% were motoneurons, 27% nonmotoneurons, and 9% multitarget neurons, whereas in the NOS‐IR population, 57% were motoneurons, 39% nonmotoneurons, and 4% multitarget neurons [33]. Therefore, more than half of the myenteric neurons function as motoneurons in the guinea pig stomach. If the concept of the multifunctionality of MEN also applies to the stomach, it is likely that at least some of the gastric MEN belong to this large group of motoneurons, directly projecting to the muscle layers and not to second‐order neurons. The question arose of whether MEN project to neurons situated in ganglia not directly adjacent to the stimulated ganglia, but even farther away. Previous studies showed that in the guinea pig stomach, the length of ChAT‐IR nonmotoneurons varies between 0.1 and 2.8 mm with a median of 0.7 mm, and the length of NADPH‐positive nonmotoneurons (which colocalize with NOS‐IR neurons [65]) ranges between 0.3 and 3.5 mm, with a median of 0.6 mm [33]. In another study, 11% of the interneurons exhibited short projections within a 1 mm radial area, most of the interneurons (65%) projecting within a radial area of 1.0–4.5 mm, and one fourth of the interneurons in the guinea pig stomach exhibited long projections in a radial area over 4.5 mm [35]. Based on this, we can state that at least some of the neurons possibly project to neurons in the directly neighboring ganglia. This includes the small group of interneurons with short projections of less than 1 mm, as well as an indefinable proportion of the largest group with projection lengths between 1 and 4 mm. However, a quarter of the interneurons described by Schemann et al. appeared to project to targets farther away than just the neighboring ganglia [35]. These observations lead us to hypothesize that the majority of MEN is likely to be multifunctional and to directly project to the muscle.

## 9 Extrinsic Innervation in the Stomach and Its Connection With the ENS

The gastric extrinsic innervation is mostly parasympathetic and dominated by the vagus nerve. The vagus nerve originates from the medulla oblongata, specifically from the dorsal motor nucleus of the vagus and the nucleus ambiguous. It has two main branches relevant for gastric innervation: the anterior (left) trunk and posterior (right) vagal trunk, which arise from the esophageal plexus as they pass through the diaphragm. The anterior vagal trunk mainly supplies the lesser curvature of the stomach, including the antrum and pyloric region. The posterior vagal trunk provides branches to the greater curvature and the fundus of the stomach. The vagal branches contain both afferent and efferent fibers, albeit the afferent fibers clearly outnumber the efferent ones (~75%). With retrograde tracing methods different kinds of afferent terminals have been described: for example, the low threshold vagal mechanosensors called “intraganglionic laminar endings” [66]. Within the stomach wall, the efferent vagal fibers synapse with myenteric neurons. They release ACh, activating nicotinic receptors on the myenteric neurons. The majority of myenteric neurons receive fEPSPs after stimulation of the vagus nerve. These were abolished by perfusion with the nicotinergic receptor blocker hexamethonium [67].

Sympathetic fibers innervating the stomach originate from the thoracic segments (T5‐T9) of the spinal cord. These preganglionic fibers travel through the sympathetic chain without synapsing and form the greater splanchnic nerves. The greater splanchnic nerves synapse in the celiac ganglion, a major prevertebral ganglion located near the origin of the celiac artery. From the celiac ganglion, postganglionic sympathetic fibers travel along the blood vessels to reach the stomach. These sympathetic fibers release noradrenaline, which can bind to alpha‐2 (α2) presynaptic adrenergic receptors, inhibiting myenteric neuronal activity.

## 10 Gastric Reflex Pathways: Differences to the Intestine

The existence of polarized innervation pathways for the circular and longitudinal muscle layers in the gastric wall [33, 35, 36, 68] seems to indicate the existence of hardware circuits, which (similar to the small intestine) initiate ascending cholinergic excitatory and descending nitrergic inhibitory reflexes. These reflexes include prominent interneuronal connections (most myenteric neurons receive cholinergic input mediated by nicotinic ACh receptors) and are abolished by hexamethonium [44, 69]. However, there are substantial differences between the polarized innervation of the stomach and the small intestine. These are likely based on the different functions of the two organs: storage and mixing versus resorption and aboral propulsion. Gastric ascending neurons outnumber descending ones, which is not the case in the ileum, and the gastric longitudinal muscle layer receives a prominent inhibitory innervation, which seems to be critical for the specific motor pattern of the stomach [35, 70]. In the stomach, longitudinal and circular muscle layers are synchronously activated to regulate muscle tone for accommodation or propulsion reflexes, while in the small intestine when the circular muscle contracts, the longitudinal muscle only exhibits a passive elongation [71]. Interestingly, in the stomach, the two muscle layers show different contractility properties, which are likely reflected by the stomach motility not only after neurogenic but also after myogenic activation [72]. Even structurally, great differences exist between the stomach and the intestine: In the stomach, the submucosal plexus is only rudimentarily present. Moreover, the myenteric ganglionic network does not follow the circular muscle direction as in the intestine, but has a honeycomb structure. It is likely that the gastric myenteric neurons fulfill functions which, in other gut regions, are specifically coordinated by submucosal neurons.

## 11 Concluding Remarks and Outlook

Much progress has been made in recent years to unravel the neuronal foundations of the stomach's distinctive accommodation, emptying, and motility patterns. Studies have advanced our understanding of the structural and functional roles of various cell types, such as the characterization of MEN as new players involved in gastric neuronal mechanosensitivity. However, numerous areas still require deeper investigation. Key aspects like the specific receptors that mediate mechanosensitivity in gastric neurons, the detailed involvement of ICCs in coordinating physiological gastric motor functions, and the application of modern single‐cell RNA sequencing should be parts of future studies. Further research in these areas will help refine our understanding of gastric motility and its underlying mechanisms, ultimately aiding in the treatment of gastric motility disorders.

## Author Contributions

S.M. was responsible for the design and creation of all figures included in this review, ensuring clarity and visual representation of key concepts. G.M.‐W. and K.E. conceived the article and drafted the manuscript. All authors approved the final draft of the manuscript.

## Conflicts of Interest

The authors declare no conflicts of interest.

## Data Availability

The data that support the findings of this study are available from the corresponding author upon reasonable request.
